# Genome-Wide SNP Data Revealed the Extent of Linkage Disequilibrium, Persistence of Phase and Effective Population Size in Purebred and Crossbred Buffalo Populations

**DOI:** 10.3389/fgene.2018.00688

**Published:** 2019-01-08

**Authors:** Tingxian Deng, Aixin Liang, Jiajia Liu, Guohua Hua, Tingzhu Ye, Shenhe Liu, Giuseppe Campanile, Graham Plastow, Chunyan Zhang, Zhiquan Wang, Angela Salzano, Bianca Gasparrini, Martino Cassandro, Hasan Riaz, Xianwei Liang, Liguo Yang

**Affiliations:** ^1^Key Lab of Agricultural Animal Genetics, Breeding and Reproduction of Ministry of Education, Huazhong Agricultural University, Wuhan, China; ^2^Guangxi Provincial Key Laboratory of Buffalo Genetics, Breeding and Reproduction Technology, Buffalo Research Institute, Chinese Academy of Agricultural Sciences, Nanning, China; ^3^Department of Veterinary Medicine and Animal Productions, University of Naples “Federico II”, Naples, Italy; ^4^Department of Agricultural, Food, and Nutritional Sciences, University of Alberta, Edmonton, AB, Canada; ^5^Department of Agronomy Food Natural Resources Animal Environmental, University of Padova, Legnaro, Italy; ^6^Department of Biosciences, COMSATS Institute of Information Technology, Sahiwal, Pakistan

**Keywords:** buffalo, effective population size, linkage disequilibrium, persistence of phase, purebred population, crossbred population

## Abstract

Linkage disequilibrium (LD) is a useful parameter for guiding the accuracy and power of both genome-wide association studies (GWAS) and genomic selection (GS) among different livestock species. The present study evaluated the extent of LD, persistence of phase and effective population size (*Ne*) for the purebred (Mediterranean buffalo; *n* = 411) and crossbred [Mediterranean × Jianghan × Nili-Ravi buffalo, *n* = 9; Murrah × Nili-Ravi × local (Xilin or Fuzhong) buffalo, *n* = 36] buffalo populations using the 90K Buffalo SNP genotyping array. The results showed that the average square of correlation coefficient (*r*^2^) between adjacent SNP was 0.13 ± 0.19 across all autosomes for purebred and 0.09 ± 0.13 for crossbred, and the most rapid decline in LD was observed over the first 200 kb. Estimated *r*^2^ ≥ 0.2 extended up to ~50 kb in crossbred and 170 kb in purebred populations, while average *r*^2^ values ≥0.3 were respectively observed in the ~10 and 60 kb in the crossbred and purebred populations. The largest phase correlation (*R*_*P, C*_ = 0.47) was observed at the distance of 100 kb, suggesting that this phase was not actively preserved between the two populations. Estimated *Ne* for the purebred and crossbred population at the current generation was 387 and 113 individuals, respectively. These findings may provide useful information to guide the GS and GWAS in buffaloes.

## Introduction

Genomic selection (GS) has been widely used to estimate the breeding values in various fields, such as animal and plant breeding programs (Newell and Jannink, 2014; Liu and Chen, 2017; Weller et al., 2017). These breeding programs select their breeding animals or plants based on predicted genomic breeding values (GBVs). However, the accuracy of GBVs is vital for the successful application and is mainly affected by estimation methods (Vanraden, 2008), marker density (Solberg et al., 2008), linkage disequilibrium (LD) (Cañas-Álvarez et al., 2016; Lenz et al., 2017), and the training population size (Akdemir et al., 2015).

LD is defined as the non-random association of alleles at different loci in a given population. The LD extent differs among different livestock breeds and is influenced by their evolutionary history and effective population size (*Ne*). The *Ne* that is estimated using the *r*^2^ coefficient has been an explosion of interest in the application of population genetics (Wang et al., 2016) and conservation biology (Husemann et al., 2016). Notably, *Ne* can serve as an essential parameter for determining the GS accuracy in livestock species (Daetwyler et al., 2010). In this regard, several modern technologies, such as genome-wide SNP array and high-throughput sequencing created new opportunities to estimate the LD extent and *Ne* in livestock (Qanbari et al., 2010; Biegelmeyer et al., 2016) and human (Tenesa et al., 2007; Park, 2011). For instance, Cañas-Álvarez et al. (2016) reported the average *r*^2^ value of 0.20 was obtained by using only 5% (38,000 SNPs) of BovineHD chip, which corresponded to an average genomic distance of 80 kb. Existing evidence revealed that the average *r*^2^ value of 0.20 was considered enough to achieve an accuracy of >0.80 for GBVs estimation (Calus et al., 2008; Meuwissen, 2009; Brito et al., 2011). Consequently, understanding of the LD extent and *Ne* in the buffalo population is essential for the application of GS technology.

Water buffalo (*Bubalus bubalis*) is a dual-purpose (milk and meat) livestock across the world that can generally be divided into two subspecies: River (2*n* = 50) and Swamp (2*n* = 48) buffalo. These animals were domesticated 3,000–6,000 years ago, and the independent domestication events occur in swamp buffalo from China and the river buffalo from the Indian subcontinent (Lei et al., 2007). To date, the river buffalo including Murrah, Nili-Ravi, and Mediterranean buffalo breeds are mainly distributed in India, Pakistan, and Italy, respectively, while the swamp buffalo are mostly distributed in the Asian countries, with China having its largest population. The Chinese swamp buffaloes have recently been divided into 14 local types based mainly on regional distribution (Yue et al., 2013). In an attempt to improve milk production traits, exotic dairy buffalo breeds (Murrah, Nili-Ravi, and/or Mediterranean buffaloes) were imported to China in 1950s. The river buffaloes are usually selected as male parents in the crossbreeding system, while swamp (local) buffaloes or crossbred offspring are used as female parents. After multiple cross breeding for several decades, a new crossbred breed has emerged with average milk production of ~1,700 kg per lactation which is significantly lower than that of the purebred breeds (2,200 kg/lactation). Notably, to investigate the LD extent and *Ne* is essential for dissecting the economically important traits and further development of the molecular breeding technology in buffaloes. The *Axiom*® *Buffalo SNP genotyping Array* (90K) (ThermoFisher Scientific, Santa Clara, CA, USA) is the only commercial SNP genotyping array that can be utilized for obtaining the genome-wide SNP data in buffalo. Using this SNP genotyping array, moderate *r*^2^ levels (0.20–0.32) were observed in Brazilian buffalo for greater distances (10–70 kb) (Cardoso et al., 2014). However, limited information on the extent of genome-wide LD has yet been assessed in different buffalo breeds. Hence, this study aims to investigate the extent of LD, determine the persistence of phase, and estimate the *Ne* in the purebred Mediterranean breed and crossbred buffalo population.

## Materials and Methods

### Sample Collection and Genotyping

All experimental procedures and designs were approved by the Committee for the Ethics University of Naples “Federico II” Italy and Huazhong Agricultural University, Wuhan, China.

A total of 495 buffaloes, including 430 purebred Mediterranean and 65 Chinese crossbred buffaloes [Mediterranean × Jianghan × Nili-Ravi buffalo, *n* = 29; Murrah × Nili-Ravi × local (Xilin or Fuzhong) buffalo, *n* = 36], were used in the present study. The purebred was selected from four herds in the Southern part of Italy, while the crossbred animals were chosen from two herds located at the Hubei Jinniu farm and Guangxi Buffalo Research Institute, respectively. The crossbred individuals were 3-way cross buffaloes and selected by the pedigree information against full- or half-sib animals. Genomic DNA was isolated from the blood sample using the standard phenol-chloroform extraction protocol. Genotyping was conducted at the Delta Genomics (Edmonton AB, Canada) using the *Axiom*® *Buffalo SNP Genotyping Array*. Quality control (QC) was performed using PLINK v1.90 (Purcell et al., 2007) software under the following criteria: call rate ≥ 0.95, minor allele frequency (MAF) ≥ 0.05, and highly significant deviations (*P* ≥ 10^−6^) from Hardy-Weinberg Equilibrium (HWE). For the studied populations, principal component analysis (PCA) was used to estimate the population admixture using the R (Null et al., 2013) with the aim to identify the unrelated individuals (Figure S1). Finally, remaining SNPs for purebred (*n* = 411) and crossbred (*n* = 45) buffaloes after QC were included for further analysis.

### Minor Allele Frequency and Haplotype Blocks Construction

The PLINK v1.90 (Purcell et al., 2007) was utilized to calculate the MAF for each SNP in the studied population, and their results were analyzed and plotted using the in-house R-scripts.

Haplotype block structure characterizes the typical patterns of LD in populations and has immediate implications for genetic studies (Guryev et al., 2006). Here, the inference of haplotype was performed using the Expectation Maximization (EM) algorithm approach implemented in PLINK v1.90 (Purcell et al., 2007) with the default parameters.

### Linkage Disequilibrium Analysis

The LD was determined using the pairwise *r*^2^ (Hill and Robertson, 1968) and calculated for each pair of loci on each chromosome (Lynch and Walsh, 1997). The equation for LD estimate is represented as follows:

(1) $$\begin{array}{r} r^{2}=\frac{{\left(p_{AB}p_{ab}-p_{Ab}p_{aB}\right)}^{2}}{p_{A}*p_{a}*p_{B}*p_{b}} \end{array}$$

where, *p*_*A*_, *p*_*a*_, *p*_*B*_, and *p*_*b*_ are the frequencies of alleles A, a, B, and b, respectively; *p*_*AB*_, *p*_*ab*_, *p*_*Ab*_, and *p*_*aB*_ are the haplotype frequencies among the alleles in the population. The LD values for each breed were separately estimated using the genome-wide SNP data. The LD decay was then estimated for 10 kb intervals (from 0 to 1,000 kb). Three minimum MAF thresholds (MAF ≥ 0.05, MAF ≥ 0.1, MAF ≥ 0.2) were selected for calculating the effects of MAF on LD estimate.

Random sampling for the purebred population was performed by taking bootstrap subsamples of size 25, 45, 51, 55, 102, 205, and 411 for the *r*^2^ estimation, aiming to estimate the effect of samples size on LD. One thousand replicates for each sample size were generated and used for calculating the average *r*^2^ values. All procedures for each samples size were performed using the in-house R scripts.

### Persistence of Phase

Persistence of phase can be used to determine the genetic relationships among populations and the reliability of the GS across different populations (Goddard et al., 2006). In this study, the SNPs that were common to the populations were selected to estimate the LD phase with the following equation (Badke et al., 2012):

(2) $$\begin{array}{r} R_{P,C}=\frac{\sum_{\left(i,j\right)\in l}\left(r_{ij\left(P\right)}-{\bar{r}}_{\left(P\right)}\right)\left(r_{ij\left(C\right)}-{\bar{r}}_{\left(C\right)}\right)}{S_{P}S_{C}} \end{array}$$

where, *R*_*P, C*_ = the correlation of phase between *r*_*ij*(*P*)_ in the purebred (P) and *r*_*ij*(*C*)_ in the crossbred (C) population, *S*_(*P*)_ and *S*_(*C*)_ = the standard deviations of *r*_*ij*(*P*)_ and *r*_*ij*(*C*)_, respectively, and = the average *r*_*ij*_ across all SNP *i* and *j* within interval *l* for populations P and C, respectively. Pearson correlations among the positive *r*-values between the populations were estimated for 100 kb intervals (from 0 to 1,000 kb) using the in-house R scripts.

### Effective Population Size

The *Ne* was estimated using the SNeP tool (Barbato et al., 2015) based on the relationship between *r*^2^, *Ne*, and c (recombination rate) (Sved, 1971). The equation is as follows:

(3) $$\begin{array}{r} N_{T\left(t\right)}={\left(4\int \left(c_{t}\right)\right)}^{-1}\left({E\left[r_{adj}^{2}|c_{t}\right]}^{-1}-\alpha \right) \end{array}$$

where *N*_*T*_ = the effective population size *t* generations ago calculated as *t* = (2∫(*c*_*t*_))^−1^(Hayes et al., 2003), *c*_*t*_ = the recombination rate; *r*^2^_*adj*_ = *r*^2^ – (β*n*)^−1^where *r*^2^_*adj*_ = the LD value adjusted for sample size (*n* = sample size, β = 2 when the gametic phase is known and β = 1 if unknown) and α = a correction for the occurrence of mutations (Ohta and Kimura, 1971).

## Results

### Marker Statistics

After removing the duplicate and chromosomal unknown SNPs, a total of 62,716 genotyped autosomal SNPs was obtained in this study. Of them, 55,090 SNPs for purebred and 55,032 SNPs for crossbred passed quality control, respectively. Moreover, both shared 52,478 SNPs (Table S1). A summary of the SNP distribution in each population was shown in Table 1. The SNPs covered ~2.47 Gb of the buffalo autosomal genome. The number of SNPs per autosome ranged from 997 to 4,627, and the average physical distance between SNPs was 44.96 kb for crossbred and 44.94 kb for the purebred breed. Moreover, the average MAF over all autosomes was 0.29 ± 0.13 in purebred and 0.32 ± 0.12 in crossbred populations, and the purebred had the higher percentage of SNPs with MAF in the range 0.05–0.1 than that of the crossbred (Figure S2).

**Table 1:** Summary of SNP distribution and average *r*^2^ between adjacent SNPs for chromosome within the crossbred and purebred populations.

**CHR^**1**^**	**Crossbred**				**Purebred**			
	**Length (Mb)**	**Number**	**Average spacing (kb)**	**Average *r^**2**^***	**Length (Mb)**	**Number**	**Average spacing (kb)**	**Average *r^**2**^***
1	201.99	4,608	43.83	0.10 ± 0.14	201.99	4,627	43.65	0.15 ± 0.21
2	188.85	4,284	44.08	0.10 ± 0.13	188.85	4,315	43.77	0.14 ± 0.20
3	175.15	3,941	44.44	0.10 ± 0.14	175.55	3,928	44.69	0.14 ± 0.21
4	165.16	3,585	46.07	0.10 ± 0.14	165.16	3,601	45.87	0.13 ± 0.19
5	127.55	2,772	46.01	0.09 ± 0.13	127.55	2,751	46.36	0.14 ± 0.21
6	120.34	2,776	43.35	0.09 ± 0.13	120.34	2,781	43.27	0.14 ± 0.20
7	117.14	2,529	46.32	0.10 ± 0.14	117.14	2,540	46.12	0.14 ± 0.19
8	119.71	2,746	43.60	0.09 ± 0.13	119.71	2,756	43.44	0.14 ± 0.20
9	109.99	2,394	45.94	0.10 ± 0.14	109.99	2,424	45.38	0.14 ± 0.20
10	104.11	2,158	48.24	0.09 ± 0.13	104.11	2,163	48.13	0.13 ± 0.20
11	102.08	2,201	46.38	0.10 ± 0.14	102.08	2,196	46.48	0.14 ± 0.20
12	106.42	2,505	42.48	0.09 ± 0.13	106.42	2,487	42.79	0.14 ± 0.19
13	90.42	1,788	50.57	0.11 ± 0.14	90.42	1,798	50.29	0.13 ± 0.20
14	82.92	1,860	44.58	0.08 ± 0.12	82.92	1,862	44.53	0.13 ± 0.18
15	82.04	1,887	43.48	0.08 ± 0.12	82.04	1,876	43.73	0.12 ± 0.18
16	84.47	1,704	49.57	0.09 ± 0.13	84.47	1,711	49.37	0.12 ± 0.19
17	72.77	1,610	45.20	0.10 ± 0.14	72.63	1,590	45.68	0.13 ± 0.21
18	65.86	1,509	43.65	0.08 ± 0.12	65.86	1,494	44.08	0.13 ± 0.18
19	71.63	1,645	43.54	0.09 ± 0.13	71.63	1,649	43.44	0.11 ± 0.20
20	68.54	1,439	47.63	0.09 ± 0.13	68.54	1,443	47.50	0.14 ± 0.20
21	60.78	1,459	41.66	0.09 ± 0.13	60.78	1,465	41.49	0.13 ± 0.19
22	61.80	1,428	43.28	0.09 ± 0.12	61.80	1,440	42.92	0.12 ± 0.17
23	51.45	1,189	43.27	0.08 ± 0.12	51.45	1,196	43.02	0.12 ± 0.19
24	42.40	1,015	41.77	0.08 ± 0.12	42.36	997	42.49	0.11 ± 0.17
All	2, 473.58	55,032	44.96	0.09 ± 0.13	2473.79	55,090	44.94	0.13 ± 0.19

*^1^CHR, the chromosome of the river buffalo (UOA_WB_1)*.

### Haplotype Block Structure

The distribution of genome-wide haplotype block within the population was shown in Table 2. After quality control, 20.04% of SNPs formed haploblocks in the purebred but only 4.71% in the crossbred population. A total of 4,557 and 722 haploblocks were detected in the purebred and crossbred populations, with the mean length of 123.08 and 82.10 kb, respectively. For the purebred population, a total of 2,564 (56.27%) haplotype blocks with the genome length >123.08 kb, and 69 blocks with the length >1.99 Mb were detected. Notably, the purebred population had the longest block coverage (560.88 Mb) in haploblock. In the crossbred population, 368 (50.97%) haplotype blocks were detected with length more than 82.10 kb, and two of them were longer than 1.99 Mb. The mean number of SNPs within the haplotype blocks was 4.36 and 3.59 for the purebred and crossbred breeds, respectively. The maximum number of SNPs within the haplotype block was 11 for purebred and 12 for crossbred, respectively.

**Table 2:** Summary statistics for haploblock structure in purebred and crossbred buffalo populations.

**Items**	**Purebred**	**Crossbred**
Blocks	4,557	722
Mean block length (kb)	123.08	82.10
Max block length (kb)	199.96	199.57
Block coverage (Mb)	560.88	59.27
SNP in blocks	11,041	2,593
BSNPs (%)^1^	20.04	4.71
Mean number of SNPs in blocks	4.36	3.59
Max number of SNPs in blocks	11	12

1 *Percentage of SNPs that form haploblocks*.

### Linkage Disequilibrium Analysis

**Figure 1:**
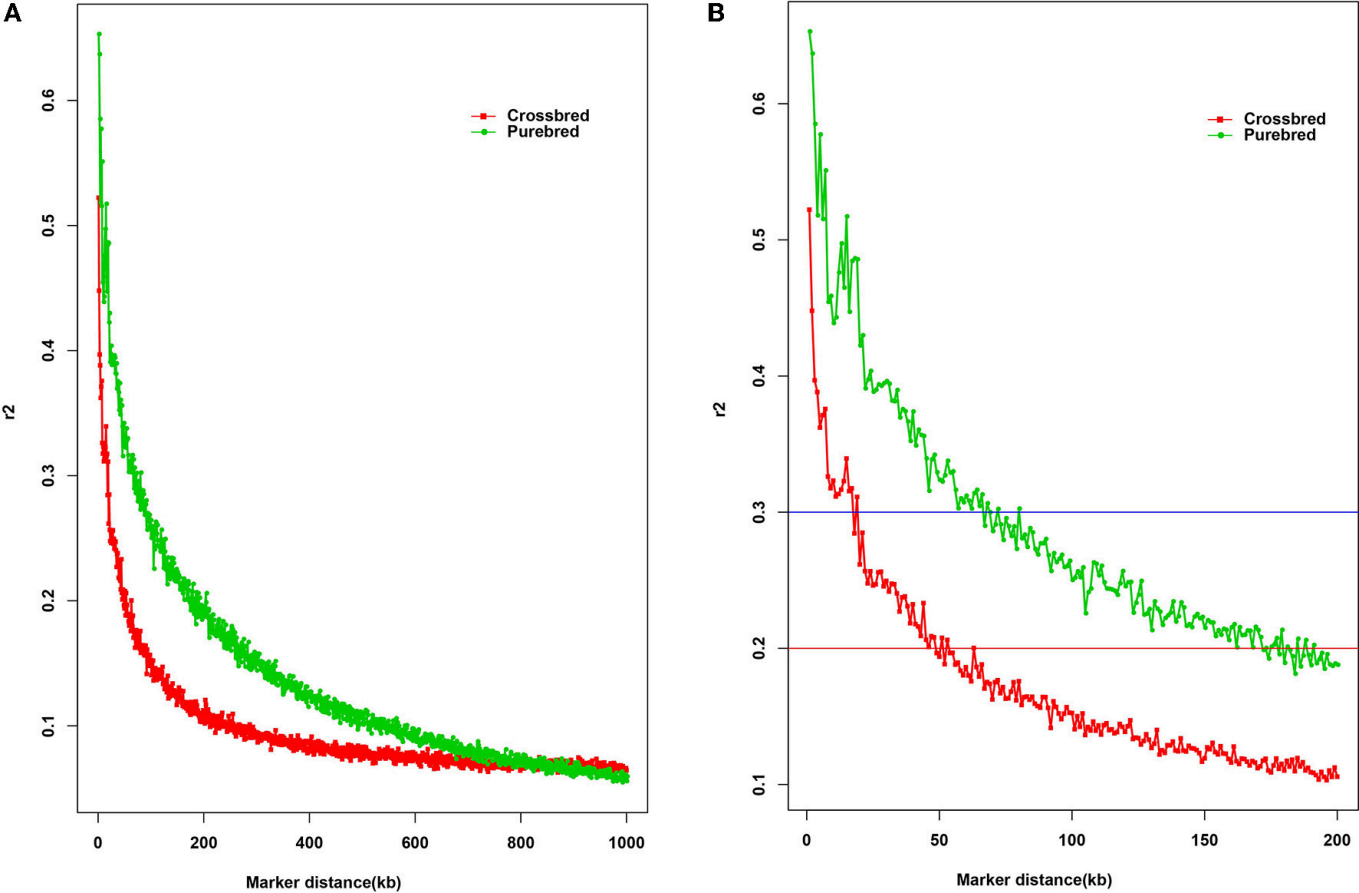
The decay of LD by distance in purebred and crossbred buffalo breeds. The solid square line with red color represents the crossbred; the solid circle line with green color represents the purebred; Red line represents the *r*^2^ value of 0.2; Blue line represents the *r*^2^ value of 0.3. **(A)** Plotted for marker distances (up to 1000 kb); **(B)** Plotted for marker distances (up to 200 kb).

LD is a fundamental approach for unveiling the genetic architecture of economically important traits in livestock species (Mckay et al., 2007). Here, the estimated overall LD in the two populations was different (Figure 1A). As expected, the average *r*^2^ in LD tended to decrease by increasing marker distance between pairwise SNPs, with a rapidly declining trend being observed over the first 200 kb. Compared with the purebred breed, the distance at which *r*^2^ decayed below 0.2 was considerably smaller in the crossbred population. Observed LD declined from 0.67 to 0.20 was ~50 and 170 kb of marker distance in the purebred and crossbred (Figure 1B), respectively. The average *r*^2^ between adjacent SNPs across all autosomes was 0.13 ± 0.19 for the purebred and 0.09 ± 0.13 for crossbred populations (Table 1). Furthermore, with *r*^2^ > 0.2, about 20.68 and 13.97% pairwise SNPs were found in purebred and crossbred. With *r*^2^ > 0.3, about 13.42 and 7.02% were found in purebred and crossbred, respectively.

**Figure 2:**
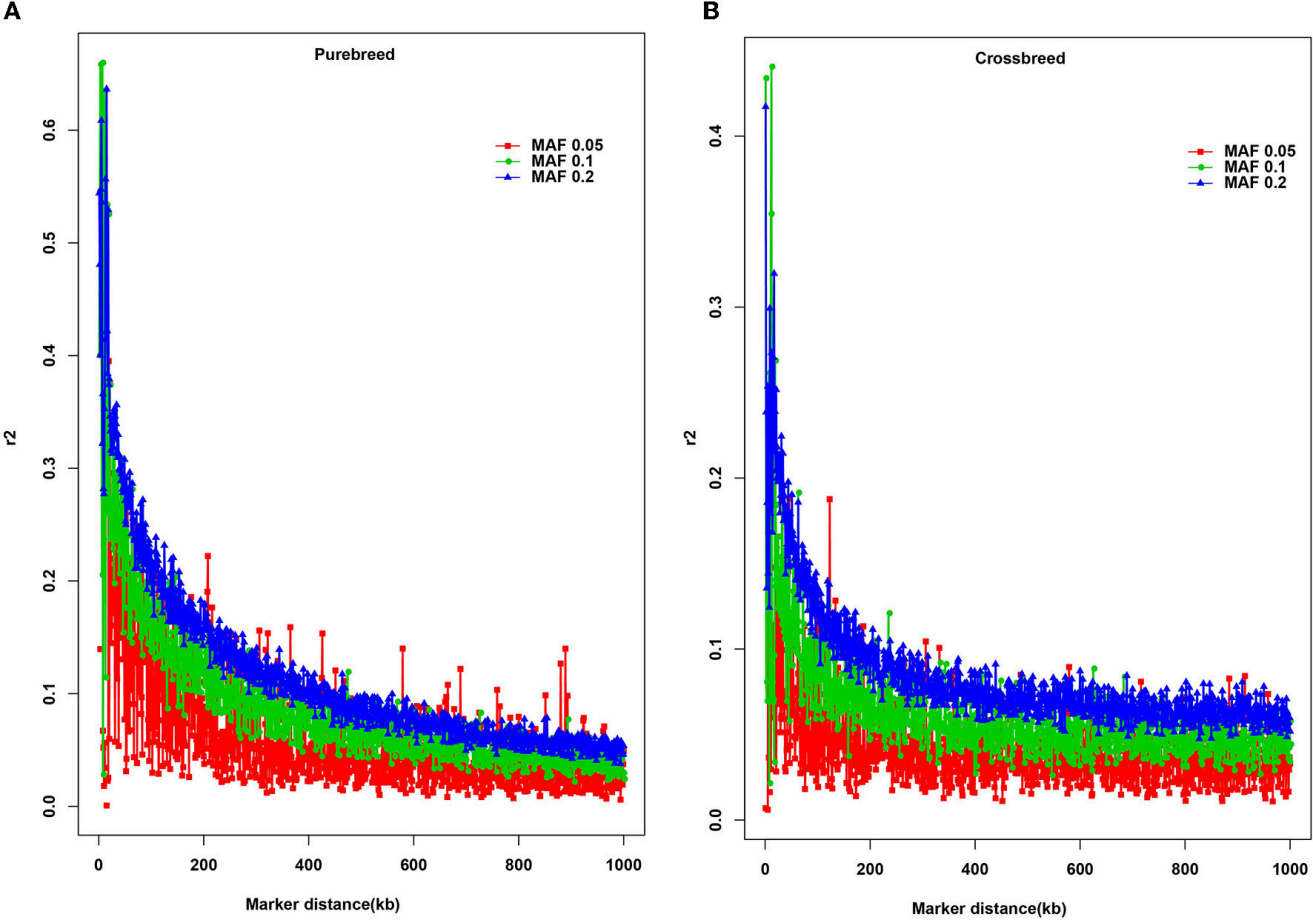
Average *r*^2^ estimates at different genetic distances for three different minor allele frequency (MAF) thresholds. The solid square line with red color represents the MAF ≥ 0.05; the solid circle line with green color indicates the MAF ≥ 0.1; the solid triangle line with blue color represents the MAF ≥ 0.2. **(A)** Average *r*^2^ estimates for the purebreed population; **(B)** Average *r*^2^ estimates for the crossbreed population.

Three different minimum MAF thresholds (0.05, 0.1, and 0.2) were selected to estimate the potential effect of MAF on the extent of LD (Figure 2). Overall, a rapidly declining trend of *r*^2^ values was found over short inter-marker distances (< 100 kb), especially the average *r*^2^ increased with MAF in the purebred (Figure 2A) and crossbred (Figure 2B) breeds.

**Figure 3:**
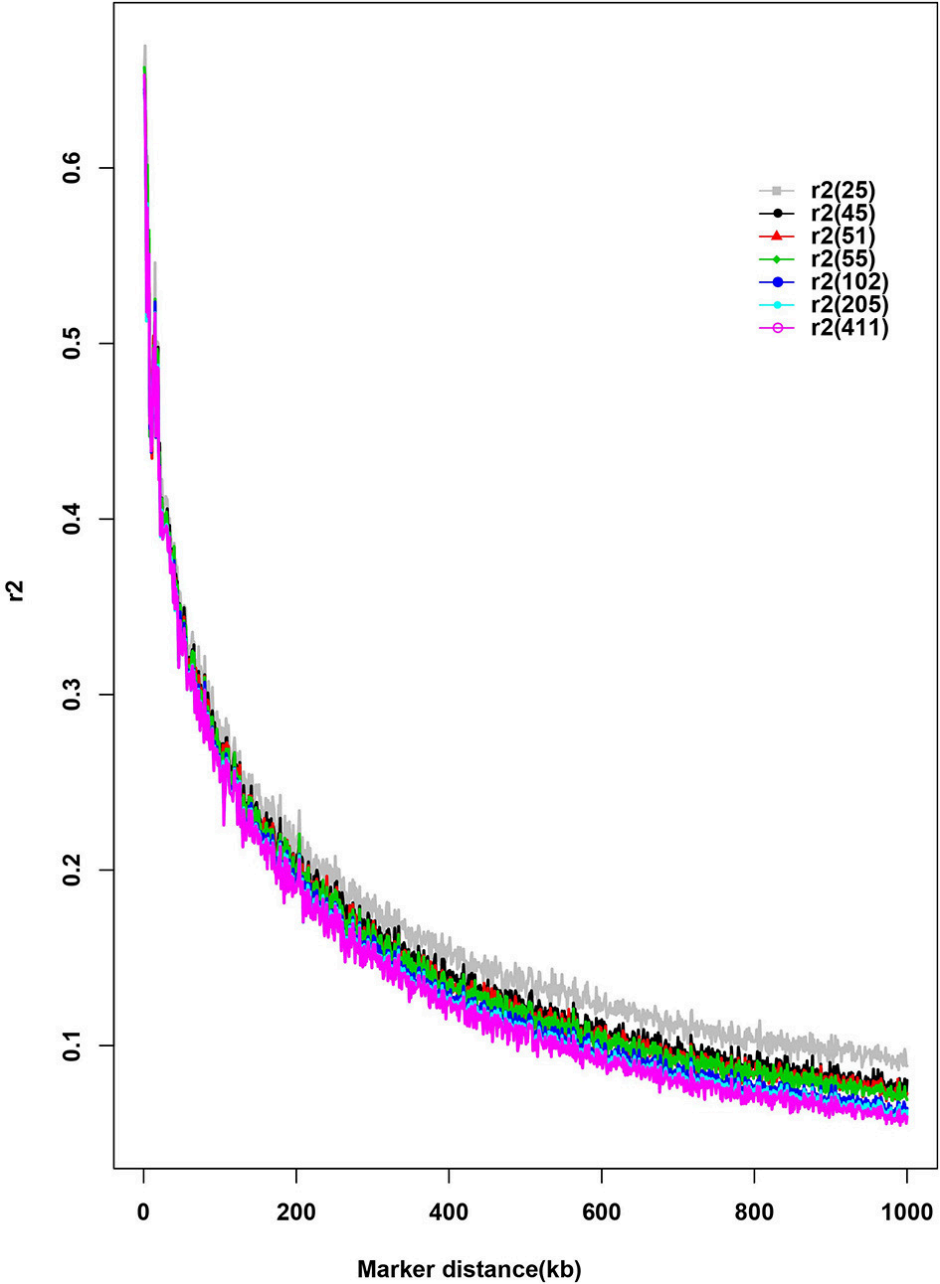
Distribution of average pairwise *r*^2^ by sample size and distance. The number in parenthesis in the legend indicates the sample size of each subset.

Seven different subsamples of purebred were used to evaluate the effect of sample size on LD estimates (Figure 3). This bias was increased with the decrease of subsample size. There was little change of LD estimates when the sample size >45. An overestimation of *r*^2^ was found when sample size was as small as 25.

### Persistence of Phase

**Figure 4:**
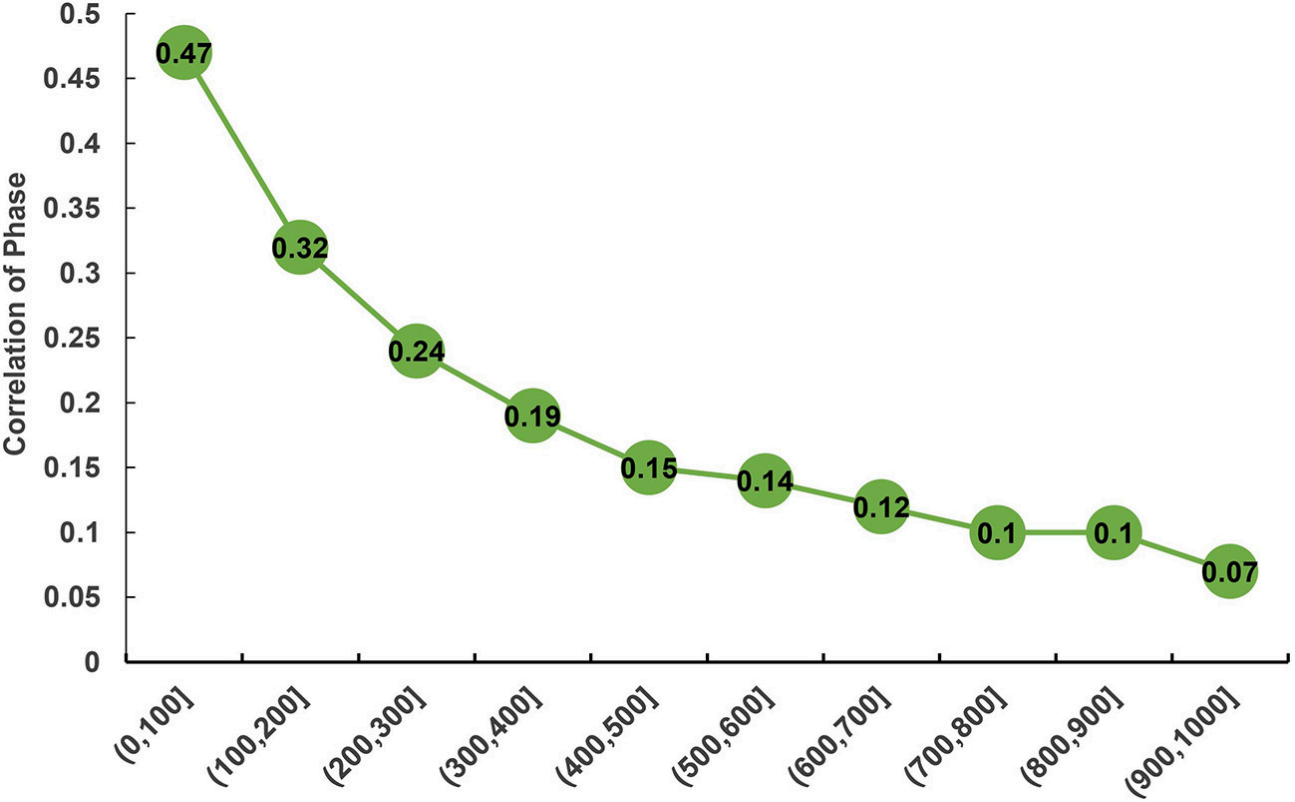
Correlation of phase between purebred and crossbred buffalo populations for SNP pairs at varying distances.

The statistic *r-*values were used to estimate the extent of persistence of allelic phase in the studied population shown (Figure 4). Overall, a declining trend was observed in the phase correlations between the breeds with an increase of distances between SNPs. The largest phase correlation (*R*_*P, C*_ = 0.47) for the two populations was observed at the distance < 100 kb, whereas the lowest correlation (*R*_*P, C*_ = 0.07) was observed at the distance of 900–1,000 kb.

### Effective Population Size

**Figure 5:**
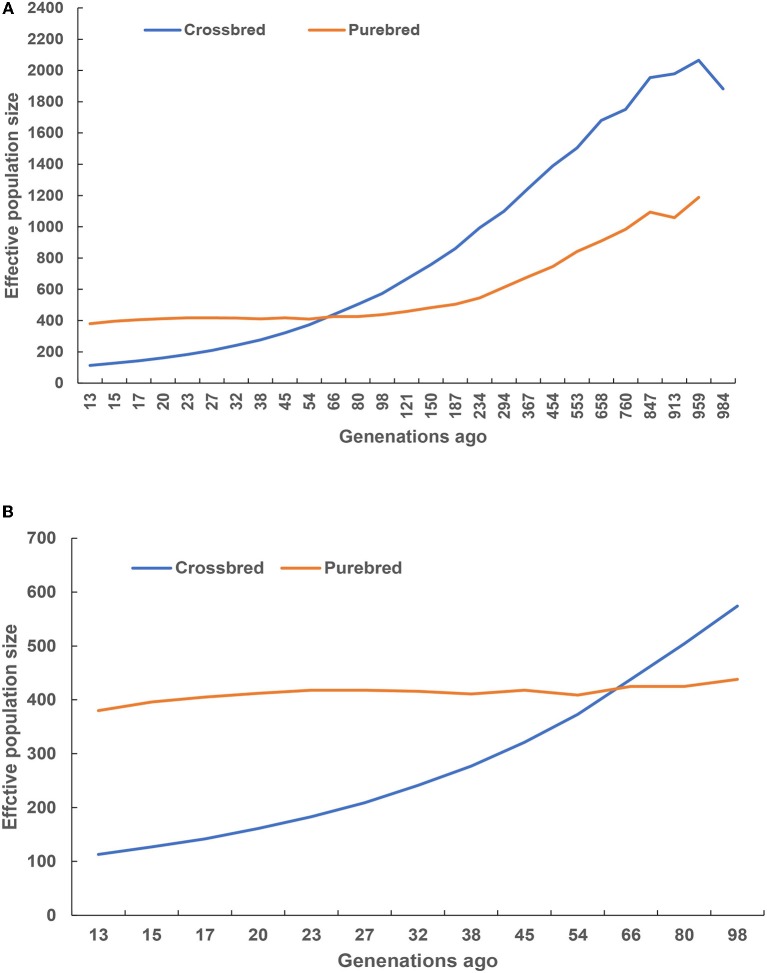
Historical **(A)** and recent **(B)** effective population size estimated using linkage disequilibrium.

Historical and recent effective population size estimates were presented in Figure 5. In total, the historical *Ne* declined from 1,000 to 100 generations ago across the two studied populations (Figure 5A). The purebred breed had higher estimates of *Ne* than the crossbred breed at 66 generations ago, and vice versa. A rapid decreasing recent *Ne* was observed in the crossbred, while the purebred had a slow *Ne* decline (Figure 5B), and their estimated values were closer to 113 and 387 at 13 generations ago, respectively.

## Discussion

To the extent of our knowledge, this is the first study to characterize the extent of LD, compare the consistency of phase, and estimate the effective population size in the crossbred and purebred buffalo populations. Numerous previous studies described the extent and pattern of LD in different domestic species by using the genome-wide SNP data (Do et al., 2014; Porto-Neto et al., 2014; Al-Mamun et al., 2015), all of which essentially increased the GWAS and GS efficiency and accuracies, and contributed to accelerate the genetic progress in economically important traits. In the present study, we utilized the Axiom® Buffalo SNP genotyping array to estimate the extent of LD, the persistence of phase and *Ne* between purebred and crossbred buffalo populations. Interestingly, a similar proportion (87.84% for purebred vs. 87.75% for crossbred) of SNPs was generated between the two populations after the QC, whereas compared to crossbred breed, a higher proportion of SNPs with MAF in the range 0.05–0.1 was found in the purebred breed. The average MAF over all autosomes was 0.29 ± 0.13 in purebred and 0.31 ± 0.12 in crossbred populations, which was slightly higher than that of MAF (0.22) in Brazilian buffaloes described by Cardoso et al. (2014). Accumulating evidence has revealed the SNPs with low allele frequencies tend to underestimate the *r*^2^ values in LD between SNPs (Qanbari et al., 2010; Espigolan et al., 2013). Therefore, three different MAF thresholds (0.05, 0.1, and 0.2) were selected and utilized to estimate the effect of MAF on the extent of LD. The results showed the average *r*^2^ of LD increased with the increase of MAF across the studies breeds, especially a rapidly declining trend of *r*^2^ values were detected at short distances (< 100 kb). Similar results were reported in other species, such as cattle (Khatkar et al., 2008) and goat (Mdladla et al., 2016). Hence, the current river buffalo SNP genotyping array can be used for the genetic studies on this species.

Previous evidence showed that small sample size (*n* ≤ 25) leads to biased estimates of LD (Khatkar et al., 2008). In this regard, Bohmanova et al. (2010) highlighted that a minimal sample size of 55 animals was required for accurate estimation of LD by *r*^2^ values. In this study, our data also demonstrated the small samples size (*n* = 25) resulted in an overestimation of *r*^2^. Interestingly, the averages *r*^2^ values for the samples with 45 individuals were consistent with that of sample size with 55 animals, implying that samples with at least 45 animals in the current study had no influence on the estimates of *r*^2^. Consequently, a lower sample size of crossbred buffalo (*n* = 45) in the present study did not affect the consistency of *r*^2^ values in both breed groups, which is consistent with the previous study by Makina et al. (2015) in four South African Sanga cattle breeds (29 ≤ sample size ≤ 54). Similarly, two different studies with a significant difference in their sample size (817 vs. 24 Thoroughbred horses) reported similar LD decay pattern as *r*^2^ values decreased from 0.6 to 0.2 when the distance between SNPs was increased to 0.5 Mb (Wade et al., 2009; Corbin et al., 2010). Therefore, we hypothesize that the sample size in our studied population (*n* = 45) might not have a significant effect on the *r*^2^ values. However, the interpretation of this inference needs to be confirmed by the large sample size of unrelated individuals.

Characterization of the haplotype block structure provides useful parameters to guide the GWAS and GS (Mokry et al., 2014). In our study, the purebred breed (4,557) had more haplotype blocks than that of the crossbred breed (722). Meanwhile, a higher mean block size with 123.08 ± 61.72 kb in the purebred was detected compared to that of the crossbred (82.10 ± 66.96 kb). We believe that these discrepancies are caused by the SNP ascertainment bias due to the procedure to design SNP array and/or the unbalanced number of purebred and crossbred buffaloes. Moreover, the crossbred population had smaller block coverage (59.27 Mb) in haploblocks. Only 4.71% of SNPs formed haploblocks in the crossbred population, compared to 20.04% in the purebred buffaloes. The finding suggested that most SNPs did not form haploblocks in the crossbred populations because of the small extent of LD. Also, these data indicated that the use of high-throughput sequencing approach was suggested to identify specific SNPs for the crossbreed buffaloes, such as Restriction-site associated DNA sequencing (RAD-seq) (Zhai et al., 2015), Specific-Locus Amplified Fragment Sequencing (SLAF-seq) (Li et al., 2017), and genotyping by sequencing (GBS) (De Donato et al., 2013). We inferred that the crossbred population in the present study were 3-way cross buffaloes with the bloodlines from the swamp and river buffaloes, but the current buffalo SNP array only provided the polymorphic SNPs across four river breeds (Mediterranean, Murrah, Jaffarabadi, and Nili-Ravi), resulting in the small haploblock size in crossbred populations.

Some critical average *r*^2^ values can be indicative of the GWAS and estimation of genomic breeding value, for example, the average *r*^2^ value of 0.3 is indicative of GWAS (Ardlie et al., 2002), while for genomic selection, *r*^2^ value of 0.2 can be served as enough to achieve an accuracy of 0.85 for GEBV (Meuwissen et al., 2001). Our study showed that at distance of 200 kb, crossbred buffalo breed showed higher rates of LD decay than the purebred breed (Figure 1B). In other words, the crossbred populations showed a small extent and rapid decay of LD by distance for all autosomes compared with the purebred population. Moreover, observed LD in the crossbred and purebred breeds decreased from 0.67 to 0.30 at ~10 and 60 kb of marker distance, respectively (Figure 1B, blue horizontal line at *r*^2^ = 0.3). Mckay et al. (2007) reported that for the whole genome association mapping, it would require 28,700 [2.87 GB/100 kb at LD (*r*^2^) = 0.2] fully informative SNPs to saturate the genome at an average resolution of 100 kb based on the bovine genome size (2.87 Gb). Here, our data suggested that a minimum of 47,000 [2.83 Gb/60 kb at LD (*r*^2^) = 0.3] SNP markers, calculated based on the buffalo genome size of 2.83 Gb approximately (Williams et al., 2017), would be needed to capture most of the LD information necessary for GWAS in two populations. The LD (*r*^2^) dropped to < 0.2 at distances between SNPs of ~50 kb in the crossbred population, whereas this drop in the purebred was observed for much greater distances (~170 kb) (Figure 1B, red horizontal line at *r*^2^ = 0.2). A similar result for the extent of LD was found between the crossbred beef cattle and purebred Angus and Charolais cattle (Lu et al., 2012). Our finding also indicated that a minimum of 16,500 SNPs [2.83 Gb/170 kb at LD (*r*^2^) = 0.2] is required for the genomic selection analysis in buffaloes. Similarly, Cardoso et al. (2014) observed moderate *r*^2^ levels (0.20–0.32) at the marker distances of 10 ~ 70 kb in Brazilian buffaloes using the buffalo 90K SNP genotyping array, which is consistent with that of our data for the purebred population. Alternately, although at least 20.68% of adjacent SNP pairs had an *r*^2^ > 0.2 and 13.42% had an *r*^2^ > 0.3 in the purebred population, only 7.02% of adjacent SNP pairs of the 90K SNP panel showed an *r*^2^ > 0.3 in the crossbred population. Therefore, our data suggested that a higher density SNP array were required for the implementation of GS in the Chinese crossbred buffalos.

Understanding the persistence of LD phase is another essential strategy for GS across breeds or population because SNPs pairs can exhibit the difference of LD phases between two populations (Goddard et al., 2006; Daetwyler et al., 2010). The correlation of the signed *r* value represents the degree of genetic relationship between two populations (de Roos et al., 2008), and determines the marker density to conduct multi-breed GS (Makina et al., 2015). Our estimates of phase correlation revealed a declining trend with increasing distance between SNPs, with the largest correlation of phase (*R*_*P, C*_ = 0.47) observed at the distance of < 100 kb. This finding indicated that the phase might be not actively preserved between breeds. Based on this finding, we assume that to increase the markers density in the array is an alternative solution for increasing the correlation of LD phase, and an adequate representation of each breed needs to be employed in the reference population when considering the application of GS in a multi-breed training population.

The *Ne* is widely regarded as one of the most critical parameters in both evolutionary and conservation biology (Charlesworth, 2009; Li and Kim, 2015), as it determines the accuracy of genomic selection (Goddard, 2009; Daetwyler et al., 2010). The current study observed the decreased pattern of *Ne* from 1,000 to 100 generations ago across the two studied population, indicating a reflection of the historical process of domestication and breed formation. Remarkably, the crossbred breed displayed higher *Ne* estimates after 66 generations ago, suggesting that these animals could have been influenced by the artificial selection. Moreover, a decreasing recent *Ne* was observed for the purebred and crossbred breeds, and their estimated values were closer to 387 and 113 at 13 generations ago, suggesting that these animals were subjected to strong selection or genetic drift that resulted in decrease population decline. However, the crossbred breeds were hybrid animals from the river and swamp buffalo subspecies, implying that these animals contained the admixture signals from hybrid genomes. In other words, the admixture signals biased estimate of the *Ne* upward throughout time (Orozco-Terwengel and Bruford, 2014). Therefore, the estimated *Ne* in the crossbred population was overestimated due to admixture signals. Alternatively, it is well-known that a small *Ne* means the reduction of genetic variation in the population, thereby hindering the genetic progress (Ni et al., 2012). In particular, *Ne* of at least 50 to 100 recommended by FAO should be maintained in animal breeding (Sørensen et al., 2005). Meuwissen and Woolliams (1994) stressed that fitness in livestock populations might decrease due to inbreeding depression. The estimated *Ne*, 13 generations ago for the purebred and crossbred buffaloes in the current study were well above the recommended numbers. However, the *Ne* slope in Figure 5B suggested that the crossbred population size was consistently decreasing, implying that actions are needed to maintain sufficiently large *Ne*, such as the reduction of wider use of artificial insemination, introducing new bloodlines from exotic river buffalo, as well as smaller progeny groups for elite sires and an increase in recorded buffalo bull numbers.

## Conclusions

In this work, our data reveal the presence of different extents of LD between purebred and crossbred buffalo populations, with purebred having highest levels of LD. Estimated *r*^2^ ≥ 0.2 extended up to ~50 kb in crossbred and 170 kb in purebred populations, while average *r*^2^ values ≥ 0.3 were respectively observed in the 10 and 60 kb in the crossbred and purebred populations. Furthermore, we observe an initial pattern of decreasing *Ne* with estimated values closer to 113 for crossbred and 387 for purebred at 13 generations ago, suggesting that the declining trend in the *Ne* of the Chinese crossbred buffaloes should be avoided, or the genetic variation in the population should be enriched by introducing new bloodlines from exotic river buffalo. Further confirmatory investigations for the crossbred breeds are required on the larger population set.

## Data Accessibility

The genotype data used in the present study are available from the Dryad Repository (doi: 10.5061/dryad.310pf05).

## Author Contributions

TD, AL, JL, MC, BG, AS, ZW, CZ, GP, and GC collected the buffalo blood samples. TD conceived and carried out the analysis, interpreted the data and wrote the manuscript. TD and LY conceived the study and participated in its design and coordination. GH, TY, SL, HR, XL, and LY reviewed the paper. All authors read and approved the manuscript.

### Conflict of Interest Statement

The authors declare that the research was conducted in the absence of any commercial or financial relationships that could be construed as a potential conflict of interest.

## Supplementary Material

Figure S1 Population structure of purebred and crossbred buffalo revealed by the principal component analysis. **(A)** The studied population before quality control filters; **(B)** the studied population after quality control filters.

Figure S2 Minor allele frequencies for SNPs that passed quality control by breeds.

Table S1 Number of SNPs genotyped and remained after quality control filters.
