# Supplementary figures and images for: Genome-Wide SNP Data Revealed the Extent of Linkage Disequilibrium, Persistence of Phase and Effective Population Size in Purebred and Crossbred Buffalo Populations
Source: Front Genet. 2019 Jan 8;9:688. doi: 10.3389/fgene.2018.00688 (PMC6332145; doi:10.3389/fgene.2018.00688)

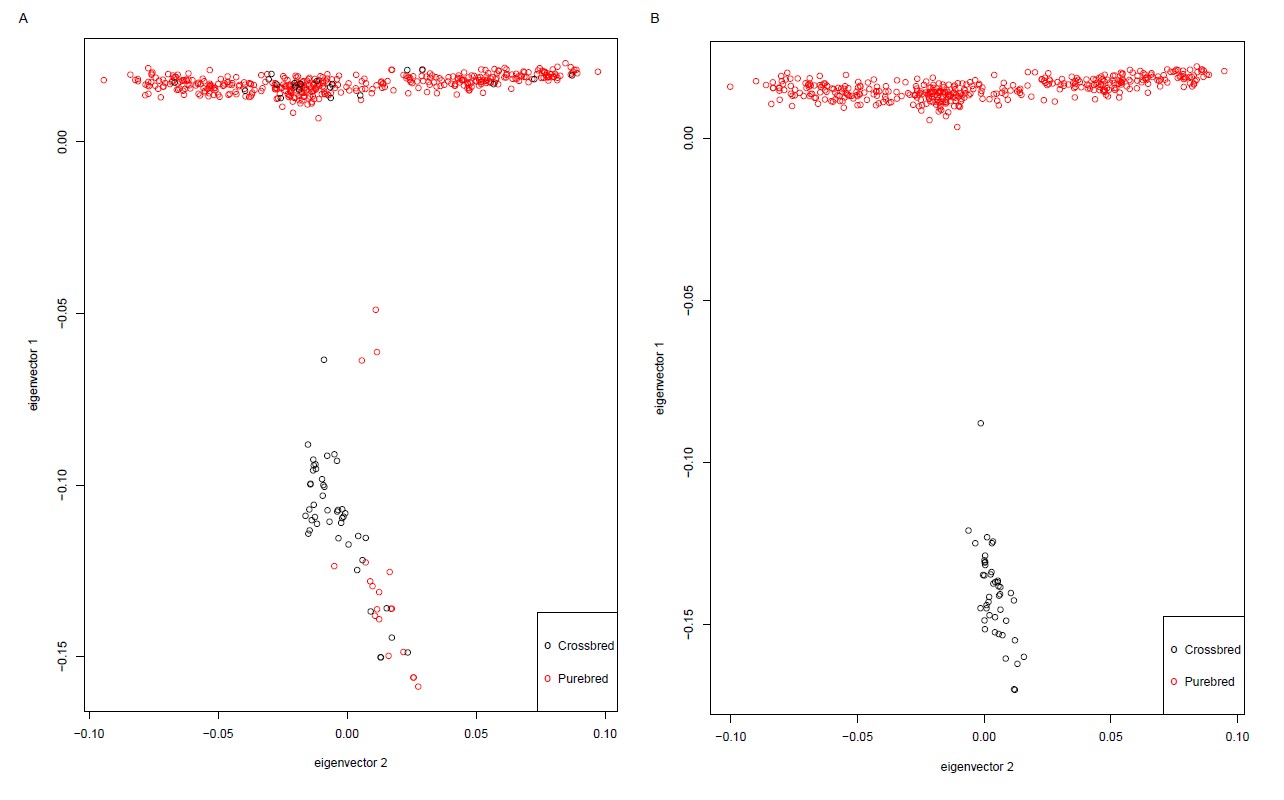

Supplement: Figure S1 — Population structure of purebred and crossbred buffalo revealed by the principal component analysis. (A) The studied population before quality control filters; (B) the studied population after quality control filters. [file Image_1.JPEG]

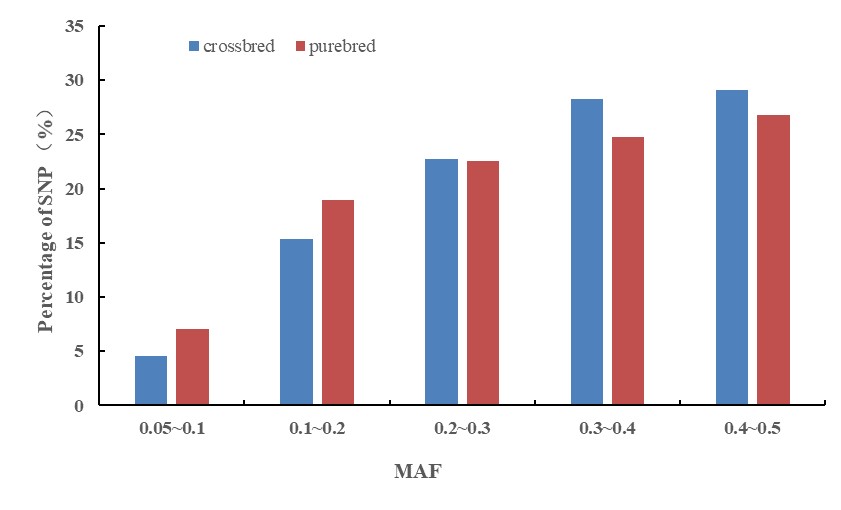

Supplement: Figure S2 — Minor allele frequencies for SNPs that passed quality control by breeds. [file Image_2.JPEG]
