# Supplementary material for: Genome-Wide SNP Data Revealed the Extent of Linkage Disequilibrium, Persistence of Phase and Effective Population Size in Purebred and Crossbred Buffalo Populations
Source: Front Genet. 2019 Jan 8;9:688. doi: 10.3389/fgene.2018.00688 (PMC6332145; doi:10.3389/fgene.2018.00688)
Supplement: Table S1 — Number of SNPs genotyped and remained after quality control filters. [file Table_1.docx]

Supplementary Material

Genome-Wide SNP Data Revealed the Extent of Linkage Disequilibrium, Persistence of Phase and Effective Population Size in Purebred and Crossbred Buffalo Populations

***Tingxian Deng^1,2^, Aixin Liang^1,*^, Jiajia Liu^1^, Guohua Hua^1^, Tingzhu Ye^1^, Shenhe Liu^1^, G. Campanile^3^, G. Plastow^4^, C. Zhang^4^, Q. Z. Wang^4^, A. Salzano^3^, B. Gasparrini^3^, M. Cassandro^5^, Hasan Riaz^6^, Xianwei Liang^2^ and Liguo Yang^1,^****

*** Correspondence:** Liguo Yang: [yangliguo2006@qq.com](mailto:yangliguo2006@qq.com); Aixin Liang: lax.pipi@mail.hzau.edu.cn

**Table S1.** Number of SNPs genotyped and remained after quality control filters.

| **Breeds**  **(N)** | **SNPs removed** | | | **Final SNPs** | **Sample size*** |
| --- | --- | --- | --- | --- | --- |
|  | **Call rate < 95%** | **HWE**  **(p < 10^-6^)** | **MAF<0.05** | **SNPs in used (%)** |  |
| Purebred  (n=430) | 1,920 | 371 | 5,335 | 55,090(87.84%)  55,032(87.75%) | 411 |
| Crossbred  (n=65) | 4,878 | 68 | 2,738 |  | 45 |
| Common SNPs |  |  |  | 52,478 |  |

*sample size passed the quality control filters from the principal component analysis and quality control analysis
